# 

**Published:** 1807-08

**Authors:** 


### NEW MEDICAL PUBLICATIONS.

A Letter containing some Observations on Fractures of the Lower Limbs; to which is added, an Account of a Contrivance to administer Cleanliness and Comfort to the Bed Ridden, &c. with Notes; by Sir James Earle. 8vo. 3s. sewed

The Edinburgh Medical and Physical Dictionary; by Robert Morris, James Kendrick, and others. 2 vols. 4to. 4l. 4s. boards.

Index Plantarum, or an Alphabetical Arrangement of all the Genera and Species of Plants hitherto described, with Reference to Original Authorities for each Genus and Species, &c. &c. by W. B. Coyt. Vol. 1, 8vo. 20s. boards.

---

### MEDICAL OBITUARY.

At his house in Old Broad Street, aged 49, William Hamilton, M. D. one of the Physicians to the London Hospital.

In New-street, Spring Gardens, aged 76, John Wasdale, M. D. formerly of Carlisle.

At the Lodge, Hillingdon, Robert Freeman, Esq. M. D. aged 73.

East Indies.

At Bundlecund, Mr. Springal Jones, Assistant Surgeon.

At Dinapore, John Finn, M. D. Assistant Surgeon.

At

At Fort William, Mr. George Rixon, Assistant Surgeon; and Mr. W. H. Turner, Assistant Surgeon.

At Chatterpore, Mr. Michael O'Donaghoe, Assistant Surgeon.

At Serringapatam, Mr. Benjamin Humpage, Surgeon.

At Manantoddy, Mr. Richard Stone, Surgeon.

At Bombay, Mr. Gabriel Alvares, Assistant Surgeon; and Mr. Jones Grisdale, Surgeon.

At Sesoor, Mr. James Guild, Assistant Surgeon.

At Tannah, Mr. Archibald Little, Surgeon.

At Surat, Dr. Mc Kenzie, Surgeon in the East India Company's service.

At Calcutta, George Boyd, Esq. Principal Surgeon on the Bengal Establishment; and Mr. Andrew Stephens, Surgeon.

At Dinagapore, Mr. William Roe, Assistant Surgeon on the same Establishment.

At Prince of Wales's Island, Henry Williams Rumsey, Esq. M. D. aged 26, in the Hon. East India Company's service at that Presidency, of a contagious fever.

---

### To CORRESPONDENTS.

We are much obliged to our numerous correspondents who have furnished us with additional proofs of the prophylactic powers of vaccination, and the consequently great decrease of mortality from the small-pox; but as the Report of the Royal College of Physicians of London, which comprise those of Dublin and Edinburgh, as well as those of the Royal Colleges of Surgeons of these cities, is now in the hands of all our readers, we think it unnecessary to publish particular reports.

We have also received, from our facetious correspondent, an anticipation of the observations which are to be expected from the *three noted, surviving, out-standing,* Antivaccinists, in their next publications, respecting the Report of the Royal College of Physicians. He takes it for granted that they will not suffer a verdict so decidedly against them, and delivered from such an authority, to pass without some notice in their usual style. This style he has attempted to imitate, and, as we think, with wonderful success. The diffidence, retiring modesty and humility of the little Doctor; the brilliant wit, sprightly vivacity, and logical acumen of the great Surgeon; the classic elegance, the refined polish, and the delightful perfume of the style of the great Doctor, are most happily pourtrayed; *sed nunc non erat his locus.*

The second part of Dr. Bellamy's case would have been published some months ago, but it has been unaccountably mislaid; we therefore request him to send us another copy.

Communications are received from Mr. Dinmore, Dr. Coxe, Dr. Kinglake, Mr. Okes, and Dr. Harrison.

